# A document level neural model integrated domain knowledge for chemical-induced disease relations

**DOI:** 10.1186/s12859-018-2316-x

**Published:** 2018-09-17

**Authors:** Wei Zheng, Hongfei Lin, Xiaoxia Liu, Bo Xu

**Affiliations:** 1 College of Computer Science and Technology, Dalian University of Technology, Dalian, China; 2 College of Software, Dalian JiaoTong University, Dalian, China

**Keywords:** Chemical-induced diseases, Document level, Knowledge, Attention mechanism, Neural network, Text mining

## Abstract

**Background:**

The effective combination of texts and knowledge may improve performances of natural language processing tasks. For the recognition of chemical-induced disease (CID) relations which may span sentence boundaries in an article, although existing CID systems explored the utilization for knowledge bases, the effects of different knowledge on the identification of a special CID haven’t been distinguished by these systems. Moreover, systems based on neural network only constructed sentence or mention level models.

**Results:**

In this work, we proposed an effective document level neural model integrated domain knowledge to extract CID relations from biomedical articles. Basic semantic information of an article with respect to a special CID candidate pair was learned from the document level sub-network module. Furthermore, knowledge attention depending on the representation of the article was proposed to distinguish the influences of different knowledge on the special CID pair and then the final representation of knowledge was formed by aggregating weighed knowledge. Finally, the integrated representations of texts and knowledge were passed to a softmax classifier to perform the CID recognition. Experimental results on the chemical-disease relation corpus proposed by BioCreative V show that our proposed system integrated knowledge achieves a good overall performance compared with other state-of-the-art systems.

**Conclusions:**

Experimental analyses demonstrate that the introduced attention mechanism on domain knowledge plays a significant role in distinguishing influences of different knowledge on the judgment for a special CID relation.

## Background

Identifying chemical-disease relations (CDRs) are significantly crucial to improve some researches and applications in the biomedical and healthcare domains [1, 2]. For example, it can contribute to biocuration of some bioinformatics databases such as Comparative Toxicogenomics Database^1^ (CTD) [3, 4]. However, manual annotation of CDRs from literature is not only expensive but also difficult to catch up with the rapid literature growth [4, 5].

There has been currently an increased interest in exploiting computational approaches such as text-mining techniques to automatically detect relations between biomedical entities. Therefore, the BioCreative V challenge included a task on automatical extraction of CDRs from curated Medline articles (only abstracts and titles). This challenge facilitates the identification of CDRs and promotes the development of text-mining techniques. In this task, all articles were manually annotated with chemical and disease mentions, their concept identifiers-MeSH ID (the identifier in Medical Subject Headings), and true chemical-induced disease (CID) relations within the scope of an article [6]. In the CDR corpus, nearly 1/3 of all relations are described as inter-sentential CID relations [5]. Arguments of inter-sentential CID relations may cross sentence boundaries and never co-occur in the same sentence. This task remains difficult and challenging mainly because it requires recognizing inter- and intra-sentential causal relationships between chemical and disease concept identifiers (entities) rather than their special mentions (mention level) in an article.

The CID task is usually regarded as a binary classification problem. The current state-of-the-art systems [7–18] mainly use three types of methods: the traditional machine learning (ML) method, the rule-based method and the deep learning (DL) method. On the whole, those systems with a combination of knowledge bases (KB) and textual information outperform ones with textual information alone in performance. The importance of background knowledge in natural language understanding has been recognized [19–24]. Leveraging external knowledge to improve performances of natural language processing (NLP) applications attracts more and more researchers. In this work, what we are interested in is how to integrate knowledge bases with texts together to effectively learn the semantic representations of an article and improve performances of a DL-based CID system.

With the recent advances in deep learning technologies, the neural-network (NN) based systems in many NLP tasks, such as question answer, relation extraction and entity recognition, have obtained good performances due to the adaptively automatically learning capability for text representations. However, few systems exploit NN approaches to perform the CID task. Only the CNN-based mention level system [17] used knowledge from CTD and improved their F-score by 13.2%. In addition, only two systems [12, 13] without KB applied convolution neural network (CNN) and recurrent neural network (RNN) to extract sentence level CID relations, respectively.

Most of systems [7–9, 11, 18] exploit traditional ML-based approaches such as support vector machine (SVM). Take the top-ranked system [9] during the BioCreative V evaluation for an example, its F-score changed from 50.73 to 67.16% after exploiting features from four types of knowledge bases including MeSH, Side Effect Resource (SIDER), MEDication Indicaton Resource (MEDI) and CTD. Similarly, Pons et al. [8] made use of a graph database which contains entities and relations from (curated) structured databases (UniProt, CTD and UMLS) and from scientific abstracts. In addition to using knowledge features derived from some databases, these systems also extracted the sentence level and document level features. The sentence level features derived from a sentence usually include various lexical and syntactic features. The document level features related to chemicals and diseases often consist of information of relevant sentences, statistical features, high-frequent entities and trigger words. Besides the SVM-based systems, the rule-based system [10] achieved competitive performances. This system built a disease dictionary derived from MeSH, the disease ontology and Wikipedia. Furthermore, the system [7] combining the advantages of rule- and ML-based approaches not only used features from CTD but also augmented their training data from existing curated data of the CTD-pfizer collaboration. However, since these systems depend on specialized designs of domain experts for features or rules, it is difficult to generalize them to other relation extraction tasks.

In summary, one of the reasons for good performances of the above all systems with KB in the CDR task may be due to the direct or indirect exploitation of CTD. In these systems, chemical-disease relationships from CTD serve as features during machine learning. CTD provides four types of manually curated chemical-disease relations which often are called as knowledge in the subsequent sections.

However, whether SVM-based systems or NN-based systems, they all didn’t distinguish the effects of different knowledge on the CID judgement. SVM-based systems [7–9, 11] took advantages of knowledge either as features of equal importance or as Boolean features, while the NN-based system [17] concatenated one-hot representations of knowledge as a feature of the model indiscriminately. Because these relations in CTD are in nature different from each other, it is impossible for them to make the same contribution to assisting a classifier to recognize a CID relation. Therefore, a system employing chemical-disease relations from CTD should make a distinction between the influences of different knowledge on identifying a special CID according to the semantic meaning of an article. Accordingly, its model should learn the representations of texts and knowledge in a way of interdependence rather than in isolation.

In this work, because of the above mentioned two reasons, we explored the issue of how to distinguish the influences of different knowledge on the judgment of a special CID relation when knowledge is used as features to incorporate into a NN-based model. Currently, attention-based models have shown great success in many NLP tasks such as question answering [24, 25], machine translation [26, 27] and relation extraction [28–30]. In the context of relation classification, by learning a scoring function to weigh concerned feature representations, attention mechanism allows a model to pay more attention to the most influential representations for a relationship category. Thus, the different knowledge from CTD may be weighed by a scoring function depending on the semantic representation of an article. Consequently, mutual influences between texts and knowledge can be revealed because of the exploiting of attention mechanism.

Overall, the contributions of this work are as follows. (1) We proposed an effective document level model incorporated domain knowledge to detect CID relations from biomedical articles. (2) A knowledge attention depending on the learned semantic representation of an article was proposed to distinguish the influences of different relations from CTD on identifying a special CID. On this basis, the final representation of knowledge was formed by aggregating weighed relations. (3) The high level representations of an article and knowledge were further weighted to evaluate their importance to final classifying results.

The experimental results on the CDR corpus demonstrate that the proposed system integrated KB are highly competitive compared with other state-of-the-art CID systems in spite of the use of less features. Moreover, experimental analyses indicate that the introduced attention mechanism on knowledge may not only distinguish the influences of different knowledge on recognizing special CID relations but also improve the performances of the proposed system.

## Methods

In the section, text processing adopted in the proposed system is first introduced. Next, an overview of the network architecture is shown. Then, the hierarchical document level sub-network module and knowledge with attention mechanism are described in detailed, respectively.

### Text processing

Appropriate text processing in NLP tasks may generally improve performances of a system to some extent. In the proposed model, the following processing operations were applied to articles of all datasets. Numbers (integers and decimals) without letters were transformed into a special token. The MeSH ID of a disease (or a chemical) substituted for the corresponding mentions. In addition, since each candidate entity often occurs multiple mentions in an article, it is crucial for a document level model to distinguish between candidate entities and other tokens of an articles to pick up the contexts more specifically. Therefore, special marks were employed to indicate the mentions of different candidate entities. For example, in the replaced sentence “The precipitating cause of ds_d012640 was believed to be a *ds_start* ds_d062787 *ds_end* of *ch_start* ch_d014148 *ch_end*”, substrings “*ch*” and “*ds*” are used to distinguish between the chemical and the disease; substrings “d014148” and “d062787” are MeSH IDs of the replaced chemical and disease, respectively; substrings “*_start*” and “*_end*” represent the beginning and end of each candidate entity, respectively. Finally, each article was divided into sentences and each sentence was parsed by our improved Standford CoreNLP Tool [31] to get the PoS (Part of Speech) tag of each word.

### Network architecture

**Fig. 1:**
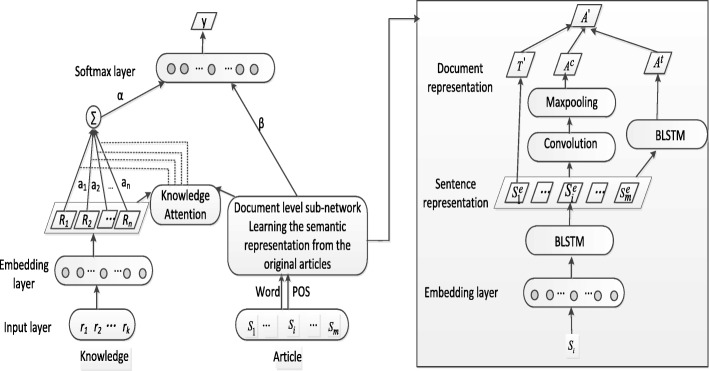
The overall architecture of the proposed model

Both knowledge representation derived from CTD and the semantic representation learned from an article will play an important role for judging the relationship of a special candidate pair. Therefore, a model should have the ability to discern which knowledge is more influential to the considered pair when it learns the semantic meaning effectively and automatically from the original text segments. Moreover, the two types of representations might have the different effects on the recognition of a chemical-disease relation. On these grounds, Fig. 1 gives an overview of the network architecture. Each article is inputted to the proposed model by sentence sequences. The main layers of the proposed model are as follows: (1) the document level hierarchical sub-network to learn the basic semantic meaning of a candidate pair only from the original text segments of an article , which is implemented by learning the semantic representation of each sentence, relations among sentences and the theme of an article; (2) the embedding layer to look up the knowledge embedding vocabulary to encode relations of CTD into vectors; (3) knowledge attention to act the semantic representation of an article on the different knowledge candidates to highlight the most influential relations for the candidate pair; (4) weighted relations to be aggregated to serve as the final knowledge representation for a given pair; (5) representations of texts and knowledge to be weighted to reflect their different effect on final classifying results; (6) the softmax layer to conduct relation classification according to the above combined semantic meanings.

#### Input representations

Given an article with n_1_ sentences $D=\left\{{S}_1,{S}_2,\dots, {S}_i,\dots, {S}_{{\mathrm{n}}_1}\right\}$, each sentence ${S}_{\mathrm{i}}=\left\{{w}_1,{w}_2,\dots, {w}_{\mathrm{j}},\dots, {w}_{{\mathrm{n}}_2}\right\}$has a maximum of n_2_ words. Since word embedding [32] maps words to low-dimensional real space where semantic meanings of words can be represented by vectors, the embedding layer of the proposed model will look up the embedding vocabulary to perform this transformation process according to the corresponding index of each input token. Here, each embedding vocabulary can be initialized either by a random process or by some pre-trained word embedding vectors.

1. **Word and PoS**: In the proposed model, the semantic meaning of each word *w*_j_ is represented by concatenating the corresponding *l*_1_-dimension word embedding vector ${\boldsymbol{w}}_{\mathrm{j}}^{\mathrm{e}}$ and *l*_2_-dimension PoS (part of speech) embedding vector ${\boldsymbol{p}}_{\mathrm{j}}^{\mathrm{e}}$. The PoS feature of a word is valuable for relation classification tasks [28]. After the word *w*_j_is passed through the embedding layer, it is denoted as a new vector ${\boldsymbol{W}}_{\mathrm{j}}=\left[{\boldsymbol{w}}_{\mathrm{j}}^{\mathrm{e}};{\boldsymbol{p}}_{\mathrm{j}}^{\mathrm{e}}\right]$ ***W***_j_ ∈ *R*^*l*^ (*l* = *l*_1_ + *l*_2_) where the symbol “;” means the concatenation operation. Hence, the sentence *S*_i_ is represented as an array ${\boldsymbol{S}}_{\mathrm{i}}^{\mathrm{e}}=\left[{\boldsymbol{W}}_1,{\boldsymbol{W}}_2,\dots, {\boldsymbol{W}}_{\mathrm{j}},\dots, {\boldsymbol{W}}_{{\mathrm{n}}_2}\right]$.
2. **Knowledge:** For a pair of chemical and disease, it has at most four types of relations in CTD, namely “*marker/mechanism*”, “*theapetic*”, “*infered*” and “*null*”. Thus, knowledge about relations is denotes as $R=\left\{{r}_1,{r}_2,\dots, {r}_{\mathrm{k}},\dots, {r}_{{\mathrm{n}}_3}\right\}$ (n_3_ = 4). If the number of relations extracted from CTD is less than n_3_, the fixed-length representation will be obtained through padding with the relation “*null*”. By looking up the knowledge embedding vocabulary to obtain the *m*-dimension embedding vector ${\boldsymbol{r}}_{\mathrm{k}}^{\mathrm{e}}$ of each relation *r*_k_, knowledge *R* is denoted as an array ${\boldsymbol{R}}^{\mathrm{e}}=\left[{\boldsymbol{r}}_1^{\mathrm{e}},{\boldsymbol{r}}_2^{\mathrm{e}},\dots, {\boldsymbol{r}}_{\mathrm{k}}^{\mathrm{e}},\dots, {\boldsymbol{r}}_{{\mathrm{n}}_3}^e\right]$.

#### The document level sub-network

As the above mentioned, the CDR corpus consists of two types of CID relations: intra- and inter-sentential relations. Candidate entities in inter-sentential CID relations may occur either among the adjacent sentences or among the nonadjacent sentences. A true CID relation is recognized according to the theme of an article, regardless of whether it is an intra-sentential relation or an inter-sentential relation. The document level hierarchical sub-network is applied to adapt to these characteristics of the CDR corpus.

1. The semantic meaning of sentences and the theme of an article

Above all, the CDR corpus contains a great number of long sentences with the more complicated structure compared with corpora of the general domain. RNN [33], especially RNN with long short term memory (LSTM) units [34], has been demonstrated to suit many NLP tasks. LSTM is superior in capturing unbounded contexts due to the introduction of the gating mechanism, especially when it is used to model variable length of long texts. However, the LSTM’s hidden state *h*_t_ collects contexts only from the previous words (the past) and knows nothing about the subsequent texts (the future). Therefore, for the sentence *S*_*i*_ of an article, the proposed model makes use of a bidirectional LSTM (BLSTM) which is composed of forward and backward LSTM. BLSTM can capture past and future contextual informaiton of the current word. Hidden states ($\overleftarrow{{\boldsymbol{h}}_{{\mathrm{n}}_2}}$and$\overrightarrow{{\boldsymbol{h}}_{{\mathrm{n}}_2}}$) of the two LSTMs at the last time step n_2_ are concatenated to form a new vector ${\boldsymbol{S}}_{{\mathrm{i}}^{\prime }}=\left[\overrightarrow{{\boldsymbol{h}}_{{\mathrm{n}}_2}};\overleftarrow{{\boldsymbol{h}}_{{\mathrm{n}}_2}}\right]$ which is regarded as the represention of the sentence *S*_*i*_. Thus, all sentences of the document *D* are denoted as an array ${\boldsymbol{D}}^{\mathrm{e}}=\left[{\boldsymbol{S}}_{1^{\prime }},{\boldsymbol{S}}_{2^{\prime }},\dots, {\boldsymbol{S}}_{{\mathrm{i}}^{\prime }},\dots, {\boldsymbol{S}}_{{{\mathrm{n}}_1}^{\prime }}\right]$.

In addition, the theme of an article is expressed by the semantic meaning of the title of the article which is usually a sentence. Likewise, utilizing the BLSTM network learns the representation ***T***^'^of the theme of an article.

(2) The semantic meaning of an article for a given pair

Furthermore, two types of sub-networks are constructed on the representation ***D***^e^ of all sentences to capture the document level semantic meaning of a given candidate pair within the scope of an article. The one is the BLSTM network on all sentences, which captures the temporal-based dependency ***A***^t ^among nonadjacent sentences. The other one is the CNN network on all sentences, which extracts local contexts among adjacent sentences. CNN is prone to capturing the local features to generate an informative latent semantic representions of text segments such as the sentence and the paragraph. In the proposed model, a convolution layer involves *f* filters which are applied to a window of *w* sentences to obtain the representation ***LC ***of local dependencies. Subsequently, a max pooling operation on the representation ***LC ***collects the global significant contexts to produce the document level representation ***A***^c^ of the candidate pair. Similar to Collobert et al. [35], the definition of the equations is as follows:

1 $$ \boldsymbol{LC}= ReLU\left({\boldsymbol{W}}_{\mathrm{c}}{\boldsymbol{D}}^{\mathrm{e}}+{\boldsymbol{b}}_{\mathrm{c}}\right) $$

2 $$ {\boldsymbol{A}}^{\mathrm{c}}=\max \boldsymbol{LC}\left(\kern0.5em \cdot, i\right)\kern1.25em 0\le i<f $$

Where ***W***_c_ is the learned matrix, ***b***_c_ is a bias vector, ***LC***(⋅, *i*) denotes the *i-*th column of the matrix ***LC****,* and *ReLU* means the rectified linear activation function. So far, for the two types of inter-sentential CIDs, the sub-network has the ability to capture the relevant contexts by exploiting the different advantages of CNN and LSTM in pattern learning.

Finally, the three document level vectors are concatenated to represent the semantic meaning of the given pair in an article, which is denoted as ***A***^'^ = [***A***^t^; ***A***^c^; ***T***^'^].

#### Knowledge with attention mechanism

Attention mechanism has been successfully applied to some NLP tasks. The CDR task requires classifying the relation between a pair of candidate chemical and disease according to the discussed topic of an article. It is obvious that not all relations of CTD have equal contributions to helping to determine the relationship type of the candidate pair. Therefore, it is necessary for each relation from CTD to learn a weight to reflect its level of effect on the final classification. Since the relation type of a given pair mainly relies on the semantic meaning of an article, acting the semantic meaning of the article on each relation from CTD may highlight which relation from CTD is the most influential for the considered pair. For this purpose, the proposed model applies attention mechanism to original knowledge vectors for weighing each relation in CTD. We exploit the item *α*_k_ of a row vector *α* to quantify the relevance degree of each relation *r*_k_ from CTD with respect to the semantic meaning ***A***^'^of an article, the related equations are defined as follows:

3 $$ {\alpha}_{\mathrm{k}}=\frac{\exp \left(s\left({\boldsymbol{A}}^{\prime },{\boldsymbol{r}}_{\mathrm{k}}^{\mathrm{e}}\right)\right)}{\sum \limits_{{\mathrm{k}}^{\prime }=1}^{{\mathrm{n}}_3}\exp \left(s\left({\boldsymbol{A}}^{\prime },{\boldsymbol{r}}_{{\mathrm{k}}^{\prime}}^{\mathrm{e}}\right)\right)} $$

4 $$ s\left({\boldsymbol{A}}^{\prime },{\boldsymbol{r}}_{\mathrm{k}}^{\mathrm{e}}\right)=\frac{{\boldsymbol{A}}^{\prime }{\boldsymbol{Wr}}_{\mathrm{k}}^{\mathrm{e}}}{m} $$

5 $$ {\boldsymbol{r}}_{{\mathrm{k}}^{\prime }}={{\boldsymbol{r}}_{\mathrm{k}}^{\mathrm{e}}}^{\ast }{\alpha}_{\mathrm{k}} $$

Here, $s\left({\boldsymbol{A}}^{\prime },{\boldsymbol{r}}_{\mathrm{k}}^{\mathrm{e}}\right)$ is the score function, ***W*** is the learned weight matrix and *m* is the dimensionality of a knowledge vector. The dot-product operation is used to perform the calculation in Eq. (4). The new representation ${\boldsymbol{r}}_{{\mathrm{k}}^{\prime }}$ of each relation from CTD is calculated by the element-wise multiplication between its original embedding vector ${\boldsymbol{r}}_{\mathrm{k}}^{\mathrm{e}}$ and the corresponding weight *α*_k_. Then, the final representation of knowledge is derived from the aggregating effect ATT_KB_Sum of all relations from CTD:

6 $$ {\boldsymbol{K}}^{\prime }=\sum {\boldsymbol{r}}_{{\mathrm{k}}^{\prime }} $$

For the sake of comparison, we still provide other two types of knowledge representations including ATT_KB_Max and ATT_KB_Con:

7 $$ {\boldsymbol{K}}^{\prime }={\boldsymbol{R}}^{\mathrm{e}}\left(\mathrm{argmax}\left({\alpha}_{\mathrm{k}}\right),\cdot \right) $$

8 $$ {\boldsymbol{K}}^{\prime }=\underset{\mathrm{k}}{con}\left({\boldsymbol{r}}_{{\mathrm{k}}^{\prime }}\right)\kern0.5em $$

Where ***R***^e^(argmax(*α*_k_), ⋅)  denotes a row of the matrix ***R***^e^ which corresponds to the relation with the maximum weight *α*_k_, and the symbol “$\underset{\mathrm{k}}{con}$” denotes the concatenating operation acting on all knowledge vectors ${\boldsymbol{r}}_{{\mathrm{k}}^{\prime }}$.

### Training and classification

The softmax layer performs relation classification for a pair of candidate chemical and disease. After weighted representations of texts and knowledge are concatenated, the new vectors *D*_*s*_ will be passed to the softmax layer. And then, the probability distribution over each category will be output.

9 $$ {\boldsymbol{D}}_{\mathrm{s}}=\left[{\beta}_1{\boldsymbol{A}}^{\prime };{\beta}_2{\boldsymbol{K}}^{\prime}\right] $$

10 $$ p\left(y=t|D\right)= soft\max \left({\boldsymbol{D}}_{\mathrm{s}}{\boldsymbol{W}}_{\mathrm{s}}+{\boldsymbol{b}}_{\mathrm{s}}\right) $$

11 $$ \widehat{y}=\arg \underset{y\in C}{\max}\left(p\left(y=t|D\right)\right) $$

Where *β*_1_ and *β*_2_ denotes weights, ***W***_s_ is a weigh matrix, ***b***_s_ is a bias vector, *t* is the label of a category, and $\widehat{y}$denotes the predicted label of a candidate pair. The training objective is cross-entropy cost function and RMSprop (Resilient Mean Square Propagation) [36] is used to update parameters with respect to the cost function.

### Post processing

The CID task is concerned with the relations between the most specific diseases and chemicals in an article. For example, the kidney disease (general/ hypernymy) vs. chronic kidney failure (special/ hyponymy), if a chemical and chronic kidney failure hold a CID relation, the chemical and the kidney disease may not been annotated as a CID relation even if they have a semantic induced relation. Only relying on machine learning automatically may result in wrong judgements. Therefore, similar to our previous work [37], if an article includes specific diseases than a disease d_i_ which does not appear in the title, extracted chemical-disease pairs with the disease d_i_ are seen as negative instances. The hypernymy/hyponymy relations among diseases may be calculated by MeSH Tree Number.

## Results and discussion

### Dataset and evaluation settings

The CDR corpus [6] consists of a total of 1500 Medline articles: 500 each for the training, development and test set. For each given article of the CDR corpus, we first constructed relation instances because each article only annotates real CID relations. Candidate pairs <chemical MeSH ID, disease MeSH ID > were generated by matching chemical and disease entities co-occurring in an article. Moreover, entities of the inter-sentential candidate pairs were limited to co-occurrance within *K* consecutive sentences to avoid selecting unlikely candidates. Furthermore, if a candidate pair hasn’t been annotated as a CID relation in a given article, it will be labeled as negative. Table 1 shows the statistics of the constructed candidate pairs.

**Table 1:** The statistics of the CDR corpus

Dataset	CID pairs	CD pairs	Inter-sentential CID pairs	Intra-sentential CID pairs
Training	1038	5432	283	755
Development	1012	5263	246	766
Test	1066	5405	303	763
Total	3116	16,100	832	2284

The column “CD pairs” represents the total number of candidate instances

Next, we combined the original training set with the development set to argument the training set due to the limited number of samples of the CDR corpus. Similar to the common training approach of samples in NN-based systems, the union set was randomly divided into 10 equal subsets, one of which was for the new development set and the others of which all were for the new training set. The test set is still original. The minimum sentence span *K* strategy (*K* = 4 based on our previous work) only was applied to the new development and the original test datasets because of the above mentioned same reason. In addition, some real CID relations filtered by this strategy were treated as false negative instances.

The performances of the proposed model were assessed by the standard evaluation measures: precision (P), recall (R) and F-score (F). Furthermore, gold standard entities of the CDR corpus were employed to objectively evaluate each related model in this task because named entity recognition has the strong effect on the classifying performances. We used Keras library with theano backend to implement the proposed model.

### The pre-training corpora of embedding vectors

With respect to the training corpus for domain knowledge, since most articles (1400) of the CDR corpus come from the related CTD-Pfizer dataset, we downloaded the package “CTD_chemicals_diseases.xml.gz”^2^ from the CTD database and extracted the corresponding chemical MeSH ID, the disease MeSH ID and their relationship for all chemical-disease pairs (2,048,652 pairs). The CTD database provides with manually curated interactions between chemical, gene and disease. After that, *TransE*^3^ implemented by Tsinghua University was used to train the extracted triples and generate the embedding vectors of entities and relations. *TransE* [38] is an effective approach when it deals with embedding a large scale knowledge graph composed of entities and relations into a continuous vector space. The proposed model only exploited relation vectors.

Articles of the bioconcepts package (bioconcepts2pubtator_offsets.gz, about 22 gigabytes) downloaded from PubTator^4^ [39] were used as the training corpus of the word representation. The training corpus of the PoS representation comes from one fifth of texts randomly chosen from the above training corpus of the word representation. The *word2vec* tool^5^ [40] was employed to train the above two corpora and output word and PoS embedding vectors, respectively.

### Hyperparameters

We tuned the hyperparameters on the new development set (the subset with the index 0) to optimize performances of the proposed model. Table 2 lists these parameters and their corresponding values used in the proposed model.

**Table 2:** Hyperparameters

Parameter Name	Value
*l*_1:_ Word emb.Size	100
*l*_2:_ POS emb.Size	10
*m*: Knowledge emb.Size	200
The number n_1_ of sentences in an article	30
The number n_2_ of words in a sentence	120
The window size *w* for CNN	5
The number *f* of filters for CNN	300
Mini-batch	8
The number of hidden units of two LSTMs	220,440
The learning rate *lr* of RMSprop	0.001
The dropout rate	0.5

**Fig. 2:**
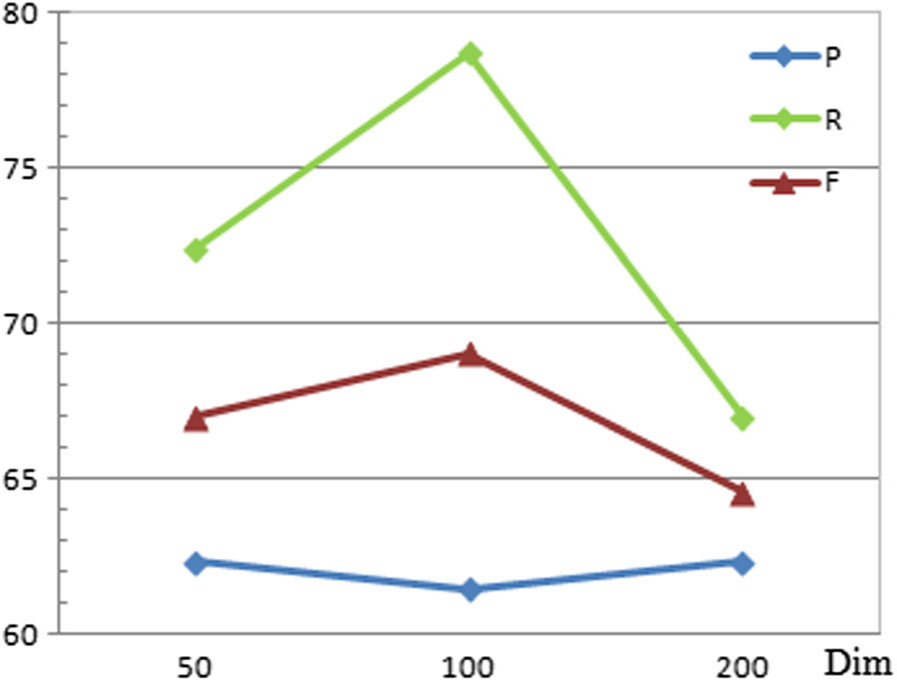
Performance evaluation for the dimension of the word embedding on the test set of the CDR corpus

**Fig. 3:**
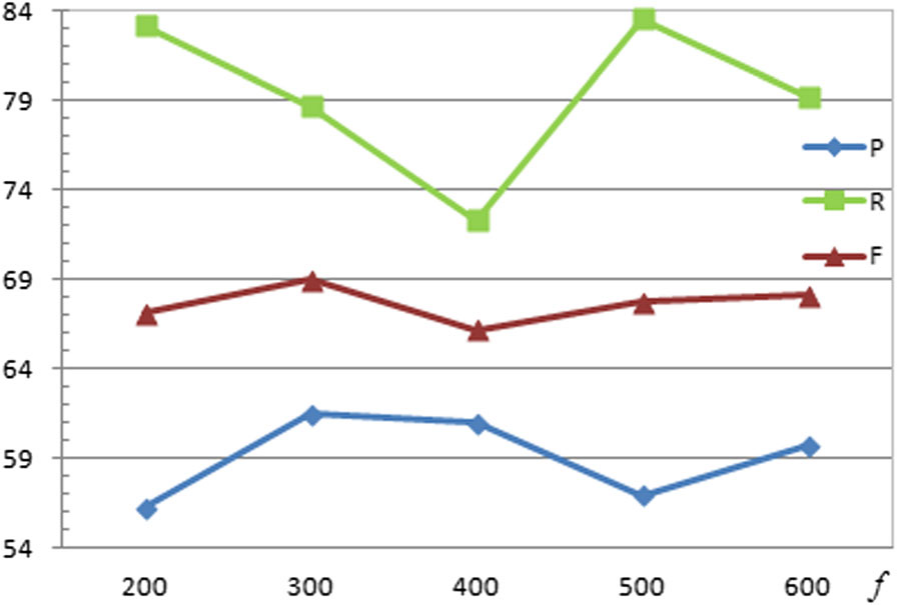
Performance evaluation for the number *f* of filters on the test set of the CDR corpus

The proposed model was tested with different dimensions of word embedding. Figure 2 shows that the 100-dimension word embedding makes the system achieve the highest F-score. The dimension of PoS embedding was set as 10 as used by Zeng [41]. Based on the statistics of CDR texts, each article includes up to 30 (n_1_) sentences and each sentence contains a maximum of 120 (n_2_) words. In addition, the evaluation for the dimension of knowledge vectors is shown in Fig. 3. The proposed system obtains the best F-score when the dimension of knowledge vectors is 200. Furthermore, two initialization methods of knowledge embedding vectors including random and *TransE* were compared to evaluate their impact on performances of the proposed system. Table 3 shows that using knowledge vectors trained by *TransE* makes the system obtain the higher precision and F-score than that by random. The reason might be due to the fact that the *TransE* method exploiting a large scale knowledge graph brings knowledge embedding vectors more targeted semantic meanings than the random method.

**Table 3:** Performance evaluation for different initialization methods of the knowledge embedding on the test set of the CDR corpus

Methods	P(%)	R(%)	F(%)
Random	57.1	81.0	67.0
TransE	61.5	78.7	69.0

The post processing step wasn’t applied to the experimental results in this table

**Fig. 4:**
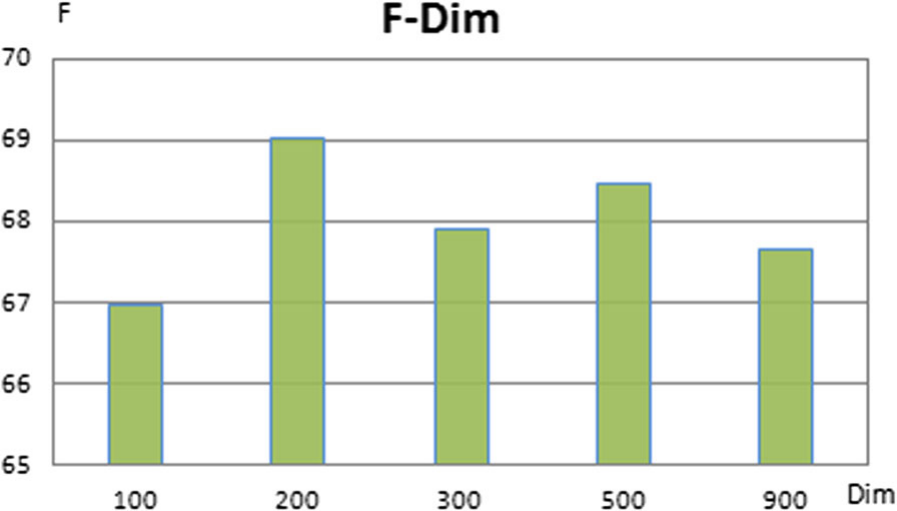
Performance evaluation for the dimension of the knowledge embedding on the test set of the CDR corpus

The numbers (220 and 440) of hidden units of two LSTM layers are equal to the size of their corresponding input dimensions in order to simplify the research process. Considering that two sentences before and after the current sentence may generally embody the semantic meaning of the inter-sentential candidate pair, we empirically set the window size w = 5. As shown in Fig. 4, the proposed system achieves a good F-score when the number *f* of filters in CNN is 300. The mini-batch was set as 8. The learning rate *lr* of RMSprop was set as 0.001 as suggested by Tieleman et al. [36]. The dropout strategy was applied on the LSTM and softmax layers to prevent the over-fitting problem, respectively. The dropout rate was assigned to 0.5 as suggested by Hinton et al. [42].

### Effects of input representations and the architecture

In NLP tasks, input features and post processing may partly influence performances of a system. Table 4 lists their effects on performances of the proposed system.

**Table 4:** Performance changes with different input representations and post processing on the test set of the CDR corpus

Feature	P(%)	R(%)	F(%)
(1): word	52.0	64.9	57.7
(2): (1) + CTD	59.5	74.8	66.3
(3): (2) + POS	61.5	78.7	69.0
(4): (3) + PP	64.4	76.7	70.0

Table 4 shows that the proposed system achieves an F-score of 57.7% when it takes only the word embedding as input. When knowledge from CTD is incorporated into the proposed model, the F-score of the system increases by 8.6%, which demonstrates that the model which integrates domain knowledge with the semantic meaning of an article may effectively promote performances of the proposed system. The effect of domain knowledge will further be analysed in the following section. Furthermore, with the introduction of the PoS feature, the precision, the recall and the F-score all are improved, which indicates that PoS tags contain a certain amount of effective information for identifying relations. Finally, post processing applied appropriately in the proposed system improves the precision and F-score to some extent.

Besides, Table 5 lists the performance changes with different components of the document level sub-network (see the right section of Fig. 1) on the test set of the CDR corpus when knowledge isn’t incorporated into the proposed model.

**Table 5:** Performance changes with different components of the document level sub-network on the test set of the CDR corpus when knowledge isn’t incorporated

Architecture	P(%)	R(%)	F(%)
(1): lstm+lstm	48.0	62.8	54.4
(2): lstm+cnn	54.8	59.2	56.9
(3): lstm+cnnlstm	56.5	61.3	58.8
(4): lstm+cnnlstm+topic	54.3	65.9	59.5

The post processing step wasn’t applied to the experimental results in this table

### Effects of knowledge with attention mechanism

1. The final representation of knowledge

Knowledge obviously contributes to the performance improvement in many NLP tasks. As mentioned above, there are four types of relations in CTD. In the proposed model, knowledge associates with the semantic meaning of an article together to perform the CID classification. Therefore, it is crucial to make the final representation of knowledge play its role more effectively. Table 6 lists different final representations of knowledge and related performances on the test set of the CDR corpus. In this table, the prefix string “ATT_KB_” denotes a model employing the proposed attention mechanism.

**Table 6:** Performance changes with the different final representations of knowledge on the test set of the CDR corpus

Methods	P(%)	R(%)	F(%)
(1): Without KB	54.3	65.9	59.5
(2): Con	61.5	74.9	67.5
(3): Sum	65.3	70.4	67.8
(4):ATT_KB_Con	59.6	79.5	68.1
(5): ATT_KB_Max	60.6	69.9	64.9
(6): ATT_KB_Sum	61.5	78.7	**69.0**

The post processing step wasn’t applied to the experimental results in this table. The highest F-score is highlighted in bold

On the whole, except for “ATT_KB_Max”, models exploiting knowledge with attention mechanism obtain the better recall and F-score than the corresponding models without attention mechanism. Compared with the approaches “Sum” and “Con” without attention mechanism, “ATT_KB_Sum” and “ATT_KB_Con” make the F-score increase by 1.2 and 0.6%, respectively. Among all approaches, “ATT_KB_Sum” achieves the best F-score. For the approach “ATT_KB_Con”, the expanded dimension of the knowledge representation derived from the concatenating operation is closer to the dimension of the semantic meaning of the article. Consequently, the redundant noise information brought by the knowledge presentation without any processing slightly weakens the learning capacity of the model. On the contrary, “ATT_KB_Sum” not only retains the proper dimension of the knowledge presentation but also highlights and fuses the most relevant knowledge representations related to a special article. This reason might also explain why “ATT_KB_Max” doesn’t achieve a relatively good performance. “ATT_KB_Max” only picks up the relation with the maximum weight as the final knowledge representation. On this basis, if an ineffective or wrong knowledge is learned, the model might partly be misled to make the wrong judgment for the relation type.

(2) Learned attention values

In addition, we manually examined the weights (attention values) of four relation types for all instances of the test set. The CID relations mainly refer to two types of relations between a chemical and a disease in the CTD task: putative mechanistic relationships and biomarker relationships. Therefore, the relation type “*marker/mechanism*” in CTD shows more obvious weight change than the other relation types because of its strong informativity. This result indicates that the type “*marker/mechanism*” makes a significant contribution to recognizing CID relations. Among the other three relation types, the relation types “*infered*” and “*null*” have the nearly weights. Accordingly, they play the minor effect on relation extraction of CID. The weight change of the type “*theapetic*” is at the intermediate level among all relation types.

**Fig. 5:**
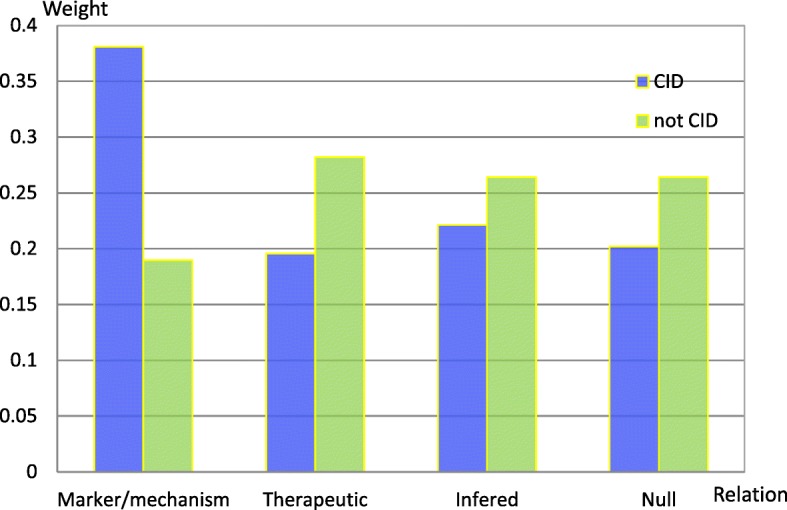
Attention value learned by the model with the approach “ATT_KB_Sum” for chemical and disease pairs

Figure 5 shows the weight of each relation learned by the proposed model with the approach “ATT_KB_Sum” for a true CID candidate (D007213 and D007022 from Doc ID 439781 in the test set) and not a true CID candidate (D009538 and D003866 from Doc ID 24114426 in the test set). The two candidate pairs contain all four types of relations in CTD. It can be seen from Fig. 5 that the relation type “marker/mechanism” has the relatively higher weight than other relation types for the true CID, while the weight of the relation type “therapeutic” is relatively higher for the not true CID. These results seem to agree with the semantic meanings of the corresponding articles. For the article containing the aboved true CID, *indomethacin* induced *hypotension* in sodium and volume depleted rats. In contrast, the article containing the above not a true CID candidate only mentions the experiments related to *nicotine* and *depression*. Hence, with respect to the recognition of the candidate relation, it might be inferred that the proposed model can learn more beneficial representations from domain knowledge bases to some extent by introducing attention mechanism targeting the document level semantic meaning of an article.

Furthermore, we assigned different weights (*β*_1_ and *β*_2_) to semantic representations of an article and knowledge. Experimental results indicate that the learned weights didn’t improve system performances. Therefore, these two values were assigned as 1 for each candidate pair.

### Performance comparisons with other systems

To evaluate our approach, we compared the proposed model mainly with the relevant models with gold standard entity annotations on the CDR corpus. Table 7 lists performances and relevant descriptions of these systems. In particular, we used each of the ten subsets as a development set and finished CID classifications on the original test dataset in turn. The average performances of ten experimental results were shown in Table 7. The standard deviation σF of F-scores is 0.67% and 0.49% before and after post processing, respectively.

**Table 7:** Performance comparisons with relevant systems using gold standard entity annotations on the test dataset of the CDR corpus

Methods	System	Methods	Text and concept level	P(%)	R(%)	F(%)
NN with KB	ATT_KB_sum	LSTM+CNN + CTD	Doc_E	60.7	78.7	**68.5**
		LSTM+CNN + CTD + pp		63.6	76.8	**69.6**
	Li et al. [17]	CNN	Doc_M	57.8	54.2	55.9
		CNN + CTD		60.0	81.5	69.1
	Verga et al. [16]	Transformer	Doc_E	55.6	70.8	62.1
		Transformer+ Extra data		64.0	69.2	66.2
Tradional ML with KB	Alam et al. [11]	SVM + CTD + pp	Doc_E + Sen_M	43.7	80.4	56.6
	Xu et al. [9]	SVM + CTD + SIDER+MEDI	Doc_E + Sen_M	65.8	68.6	67.2
	Pons et al. [8]	SVM + Graph DB	Doc_E	73.1	67.6	70.2
	Peng et al. [7]	SVM + CTD + Rules	Doc_E	68.2	66.0	67.1
		SVM + CTD + Rules +Extra data		71.1	72.6	**71.8**
	Lowe et al. [10]	rules+Ontology+WIKI+PP	Sen_M	59.3	62.3	60.8
NN without KB	Gu et al. [13]	CNN + ME+pp	Doc_M + Sen_M	55.7	68.1	**61.3**
	Zhou et al. [12]	LSTM+SVM + pp	Sen_M	55.6	68.4	**61.3**
	Gu et al. [18]	ME	Doc_M + Sen_M	62.0	55.1	58.3

The 4-th column denotes the text level and the concept level when candidate instances are constructed. “Doc” denotes the document level, “Sen” denotes the sentence level, “_E” denotes entity-based candidate pairs and “_M” denotes mention-based candidate pairs. In addition, all results listed in this table come from the corresponding improved systems after the CDR challenge. The highest F-scores  in each group of methods are highlighted in bold

These systems are divided into two groups: with KB and without KB. Obviously, most systems with KB have higher F-score than those without KB except two systems. This result further demonstrates that the effective combination of textual information and domain knowledge would improve performances of many CID systems.

For two types of CID systems including the traditional-ML-based systems and the NN-based systems, the NN-based systems can automatically learn semantic representations of text segments and domain knowledge, while the traditional-ML-based systems commonly rely on carefully handcrafted features, elaborately designed kernels and statistical features.

1. Comparison with NN-based systems

Among NN-based systems, the proposed system “ATT_KB_sum” achieves the best precision and F-score. Verga et al. [16] encoded full paper abstracts using an efficient self-attention encoder and formed pairwise predictions between all mentions with a bi-affine operation. Moreover, they improved the system performances by adding extra PubMed abstracts annotated in the CTD-pfizer dataset to their training set as Peng et al. [7] did. The chemical-disease relations from CTD were not directly applied to their system. Conversely, Li et al. [17] and our system incorporated knowledge from CTD with the semantic meaning of texts. However, Li et al. integrated knowledge only in a simple way, despite that their system achieved better performances. They used a hidden layer to covert one-hot representations of all knowledge into dense real value vectors which will be further concatenated with the semantic meaning of texts related to the nearest chemical and disease pair. They didn’t distinguish the influences of different relation types from CTD on a given chemical-disease candidate in different articles. On the contrary, attention mechanism in our system integrated the semantic representation of an article into knowledge from CTD. Thus, the importance of different knowledge with respect to a special article is discerned. Moreover, their mention level system has to define heuristic rules to determine the final relation type of a candidate pair because the CDR corpus only provides the annotations at the entity level. In contrast, we not only designed the neural network architecture at the document level but also considered the contiguity and temporality among associated sentences as well as the theme of an article.

Table 8 lists recognizing results of two types of CID relations including the intra- and inter-sentential CIDs before and after knowledge is introduced into the proposed model. It has been observed from Table 8 that, in addition to the promotion of the precision and the recall, F-scores of inter- and intra-sentential CID relations increase by 12.3 and 8.0%, respectively, after knowledge is added into the proposed model. Hence, it might be inferred that the introduction of knowledge will help to further improve overall performances of recognizing complicated inter-sentential CID relations.

(2) Comparison with tradition ML-based systems

**Table 8:** The recognizing performance of the inter-sentential and intra-sentential CIDs before and after knowledge is introduced into the proposed model

Knowledge	CIDs	P(%)	R(%)	F(%)	TP	FP	POS
Without KB	inter-sentential	45.0	42.9	43.9	130	159	303
	intra-sentential	59.5	77.9	67.4	594	405	763
With KB	inter-sentential	52.8	60.1	56.2	182	163	303
	intra-sentential	68.8	83.4	75.4	636	289	763

The experiments were performed when the new development set is the subset with the index 0 (similarly hereinafter). TP, FP and POS denotes the number of predicted true positive instances, predicted false positive instances and true positive instances of the test dataset, respectively

As shown in Table 7, NN-based systems obtain competitive performances compared with traditional-ML-based systems, most of which performed the recognition of CID relations by SVM classifier. Similar to Li et al. [17], these SVM-based systems didn’t distinguish the importance of different relations from CTD on the candidate pair of a special article. In addition to directly and indirectly utilized knowledge features, they explored a great deal of features (approximately 20 types) including entity features, various context features and statistic features. Therefore, it can be observed from Table 7 that SVM-based systems generally achieve relatively high precisions due to elaborate feature selection. On the contrary, NN-based systems exploited fewer features besides the word embedding. For example, our model only used the PoS embedding, while Li et al. only employed the position embedding. As a result, NN-based systems generally obtained relatively high recalls. However, Table 9 indicates that the proposed model has the potential for growth of the precision and F-score with the increasing number of training samples.

**Table 9:** The recognizing performance on the test dataset of the CDR corpus when different training sets were applied without postprocessing

Training set	Knowledge	P(%)	R(%)	F(%)
Only original training set	Without KB	45.2	68.1	54.3
	With KB	55.8	81.4	66.1
Train +development set	Without KB	54.3	65.9	59.5
	With KB	61.5	78.7	69.0

As for the running time, the proposed system took the server about 33 seconds to finish relation classification of CID on the CDR test set when it ran on the server equipped with 3G CPU, 125G memory and 12GB TITAN Xp GPU. Undoubtedly, the SVM-based systems run more quickly than the NN-based systems in the context of the same hardware configurations. However, with the development of hardware technologies such as processor and memory technologies, the time performance will be no longer a main problem for the classification task.

On the whole, each of traditional ML-based and NN-based systems has its advantages and disadvantages. The traditional ML-based systems not only don’t require too much training samples but also have the straightforward characteristic in the usage and the interpretability of features as well as less computational time, while the NN-based systems are able to partly automatically learn the high level representations of texts to reduce manual interventions if there are the moderate number of training samples.

## Conclusion

In this work, we proposed an effective document level neural network model integrated domain knowledge for classifying complicated relationships between chemicals and diseases from biomedical articles. Depending on the learned semantic meaning of an article, the proposed system employed attention mechanism on domain knowledge to avoid learning representations of texts and knowledge in isolation to some extent. Experimental analyses indicate that the introduced knowledge attention has the ability to distinguish the effect of different knowledge on a special candidate pair and improves performances of the proposed system. Moreover, the proposed model constructed at the document level has more advantages over sentence level or mention level models for the recognition of inter-sentential CID relations. In spite of only three types of embedding vectors, experimental results on the CDR corpus show that the proposed system achieves a good overall performance compared with other state-of-the-art systems. Furthermore, the proposed model is flexibly scalable by replacing its document level sub-network with the other high-performance sub-network modules learning the document level semantic representation of an article. Essentially, the proposed model is easy to generalize to the analogous applications integrating domain knowledge.

## Abbreviations

BLSTM: Bidirectional long short term memory
CDRs: Chemical-disease relations
CID: Chemical-induced diseases
CNN: Convolution neural network
CTD: Comparative toxicogenomics database
DL: Deep learning
F: F-score
KB: Knowledge bases
LSTM: Long short term memory
MEDI: MEDication indication resource
MeSH: Medical subject headings
ML: Machine learning
NLP: Natural language processing
NN: Neural network
P: precision
PoS: Part of speech
R: Recall
RMSprop: Resilient Mean Square Propagation
RNN: Recurrent neural network
SIDER: Side effect resource
SVM: Support vector machine

## Footnotes

1 http://ctdbase.org/

2
http://ctdbase.org/downloads/;jsessionid=9A140902CFAF161528E3DB29E4A70DCA#cd

3
https://github.com/thunlp/Fast-TransX

4
ftp://ftp.ncbi.nlm.nih.gov/pub/lu/PubTator/

5
https://code.google.com/p/word2vec/
